# Egg size and emergence timing affect morphology and behavior in juvenile Chinook Salmon, *Oncorhynchus tshawytscha*

**DOI:** 10.1002/ece3.3670

**Published:** 2017-12-06

**Authors:** Karen M. Cogliati, Julia R. Unrein, Heather A. Stewart, Carl B. Schreck, David L. G. Noakes

**Affiliations:** ^1^ Department of Fisheries and Wildlife Oregon State University Corvallis OR USA; ^2^ U.S. Geological Survey (U.S.G.S.) Oregon Cooperative Fish and Wildlife Research Unit Oregon State University Corvallis OR USA; ^3^ Oregon Hatchery Research Center Alsea OR USA

**Keywords:** growth rate, juvenile migration, maternal investment, phenotypic variation, tank orientation

## Abstract

Variation in early life history traits often leads to differentially expressed morphological and behavioral phenotypes. We investigated whether variation in egg size and emergence timing influence subsequent morphology associated with migration timing in juvenile spring Chinook Salmon, *Oncorhynchus tshawytscha*. Based on evidence for a positive relationship between growth rate and migration timing, we predicted that fish from small eggs and fish that emerged earlier would have similar morphology to fall migrants, while fish from large eggs and individuals that emerged later would be more similar to older spring yearling migrants. We sorted eyed embryos within females into two size categories: small and large. We collected early and late‐emerging juveniles from each egg size category. We used landmark‐based geometric morphometrics and found that egg size appears to drive morphological differences. Egg size shows evidence for an absolute rather than relative effect on body morphology. Fish from small eggs were morphologically more similar to fall migrants, while fish from large eggs were morphologically more similar to older spring yearling migrants. Previous research has shown that the body morphology of fish that prefer the surface or bottom location in a tank soon after emergence also correlates with the morphological variations between wild fall and spring migrants, respectively. We found that late‐emerging fish spent more time near the surface. Our study shows that subtle differences in early life history characteristics may correlate with a diversity of future phenotypes.

## 1 INTRODUCTION

Phenotypic diversity occurs widely within and across taxa. Such variation may arise simply as individual responses to variation in environmental conditions experienced throughout development (i.e., phenotypic plasticity) (see e.g., Schlichting & Pigliucci, 1998; Snell‐Rood, 2013; West‐Eberhard, 2003 and references therein). Adaptive phenotypic variation may occur as morphological differences within or across populations, including, for example, horn dimorphism as a result of larval food availability in male dung beetle (*Onthophagus taurus*; Moczek & Emlen, 1999). Additionally, we may see adaptive phenotypic diversity in behavioral traits, such as variation in spider web design based on prey availability (*Parawixia bistriata*; Sandoval, 1994), and courtship behavior variation in Threespine Stickleback (*Gasterosteus aculeatus*; Foster, 1995). The expression and variation of morphological and behavioral phenotypic traits may be driven by inheritance and early life experiences, including epigenetic and environmental factors (Schlichting & Pigliucci, 1998; Shaw & Wiley, 2010). As such, many of the differences observed in phenotypic traits later in life may have originated early on in development.

Maternal effects are a form of transgenerational epigenetics. They are defined as the genetic and nongenetic maternal contribution that can influence or modify offspring phenotypes (Bernardo, 1996; Mousseau & Fox, 1998; Wolf & Wade, 2009). In fishes, egg size is a commonly studied maternal effect because of its relationship with fecundity, maternal fitness, and offspring survival (Einum & Fleming, 2000a; Rollinson & Hutchings, 2013). However, egg size could also be influenced by genetics and maternal condition. Large eggs, which are more energetically costly to produce than small eggs, tend to yield offspring with increased survival and size (Roff, 1992; Mousseau & Fox, 1998; but see Leggett & Deblois, 1994). Egg size can vary within and across populations, as well as within individual females (Beacham & Murray, 1993; Benhaïm, Skúlason, & Hansen, 2003; Chambers & Leggett, 1996; Chambers & Waiwood, 1996; Contreras‐Sánchez, Schreck, Fitzpatrick, & Pereira, 1998; Leblanc et al., 2011; Taborsky, 2006). Subtle variation in egg size could be associated with offspring expressing different future phenotypes, and hence increase the diversity of life history characteristics in a population. Offspring from small eggs often grow faster than offspring from large eggs (Eldridge, Whipple, & Bowers, 1982; Heath, Fox, & Heath, 1999; Leblanc, 2011; Valdimarsson, Skúlason, & Snorrason, 2002). In a mouthbrooding cichlid (*Simochromis pleurospilus*), this increased growth rate in juveniles from small eggs may arise from an increase in the expression of the growth hormone receptor after hatching (Segers, Berishvili, & Taborsky, 2012). Size and growth rate in the early juvenile stage can have important implications for survival and life histories, but variation in these and other heritable and nonheritable traits can also play a role in the development of early maturation (Hutchings, 1993; Thorpe, 1986; Wirtz‐Ocaňa, Schütz, Pachler, & Taborsky, 2013). Therefore, it is important to recognize that small‐scale differences in egg size within populations may be associated with long‐term phenotypic differences and a diversity of life histories seen well after the embryonic phase of development.

During development, juveniles transition from using endogenous energy of maternal origin to exogenous feeding. The timing of depletion of this maternal energy can vary within and across populations. In salmonids, this transition from maternal energy to exogenous feeding is associated with emergence from gravel substrate. Importantly, studies have shown that hatching and emergence time may not be influenced by initial egg size (e.g., Beacham, Withler, & Morley, 1985: Chum Salmon (*Oncorhynchus keta*) and Coho Salmon (*Oncorhynchus kisutch*); Einum & Fleming, 2000b: Atlantic Salmon (*Salmo salar*); Leblanc, Kristjánsson, & Skúlason, 2016: Arctic Charr (*Salvelinus alpinus*). Like egg size, emergence timing can play an important role in promoting a diversity of life history characteristics in fishes. For example, juvenile Atlantic Salmon that began feeding only 1 week prior to others in their cohort were generally larger, more dominant, and more likely to migrate in their first year (Metcalfe & Thorpe, 1992). Thus, small variation in emergence timing may be associated with a diversity of future life history phenotypes observed within populations.

Migratory phenotypes of juvenile Pacific salmonids vary widely (Quinn, 2005). Such variation in the timing of juvenile migration may be a result of variation in adult migration. For example, salmon are often referred to as “stream‐type” or “ocean‐type” (Healey, 1983, 2001). Stream‐type adults enter freshwater substantially earlier than ocean‐type adults, will migrate further distances upstream, and spawn in headwaters, and juveniles of these stream‐type fish may rear in freshwater systems for over a year before migrating to the ocean. Conversely, ocean‐type adults tend to spawn close to the ocean in tidewaters, and juveniles migrate to the ocean within months of emergence. In addition to these large‐scale differences in juvenile migration timing across different runs of salmon, there is also variation in the timing of juvenile downstream migration within a given type or run, as seen in the Nanaimo River, Canada (Carl & Healey, 1984), the Sixes River, USA (Reimers, 1971), and the Willamette River, USA (Schroeder, Whitman, Cannon, & Olmstead, 2016), for example.

In the Willamette River basin of Oregon (OR), USA, spring Chinook Salmon (*Oncorhynchus tshawytscha*) spawn annually in the fall and juveniles emerge from gravel substrate late the following winter or early spring. Once juveniles emerge, they can distribute themselves throughout their natal tributary or the main stem river. Juveniles may also migrate to the estuary or ocean soon after emergence or at various other times up to 16 months postemergence (Schroeder et al., 2016). Importantly, these juveniles are all offspring of a “stream‐type” adult population. Juveniles that migrate in their first fall (fall migrants) tend to rear in main stem Willamette habitat compared to the tributary rearing of juveniles that migrate in the subsequent spring (spring yearling migrants). Diversity in juvenile migrant phenotypes may result from tradeoffs between survival of juveniles to the ocean and improved ocean growth conditions compared to freshwater habitats (Gross, 1987; Gross, Coleman, & McDowall, 1988; Jonsson & Jonsson, 1993; McCormick, Hansen, Quinn, & Saunders, 1998; Thompson et al., 2015). Individual size and condition could therefore influence the decision of when to undergo seaward migration (Beckman, Larsen, Lee‐Pawlak, & Dickhoff, 1998; Thompson & Beauchamp, 2014; Ward, Slaney, Facchin, & Land, 1989). Billman et al. (2014) found that fish that reared in the main stem Willamette River were similar in morphology to fish that migrated in the fall. Both were significantly different than fish that reared in upstream tributaries (McKenzie River) and fish that migrated in the following spring as yearlings, which had similar morphologies. Mainstem rearing and fall migrant juveniles had shorter heads, deeper bodies, and deeper caudal peduncles compared to the more fusiform body shape of upstream rearing and spring migrant juveniles.

These morphological differences may have resulted from environmental influences, as these fish would have reared in different river stretches; however, Unrein et al. (2017) described a difference in early life history behavior, observed at a research hatchery, that is correlated with migrant body morphologies. Soon after emergence, juveniles vertically self‐sorted themselves into surface‐oriented and bottom‐oriented phenotypes. Importantly, the surface‐oriented phenotype had similar morphology to fall migrants, while the bottom‐oriented phenotype had similar morphology to the spring yearling migrants (Unrein et al., 2017). These phenotypes were morphologically and behaviorally different despite being reared in a common environment, and there appeared to be a possible genetic link for those differences (Unrein et al., 2017). Perkins and Jager (2011) also suggested that the decision to migrate downstream as a subyearling or yearling migrant was made soon after emergence in juvenile fall Chinook Salmon from the Snake River (Idaho, USA).

In this study, we investigated the hypothesis that variation in early life history characteristics (egg size and emergence timing) will be associated with later morphological and behavioral differences in juvenile Chinook Salmon. Although we did not specifically test migration timing in our study, we investigated if the possible differences in morphology and behavior are correlated with characteristics associated with migration timing. We collected morphometric data from fish of known egg size and emergence timing to compare to the overall body morphologies of fall and spring (yearling) migrants and to known surface‐oriented and bottom‐oriented phenotypes. Salmonids that begin feeding sooner or experience faster growth rates after hatching may develop a size advantage resulting in earlier downstream migration (Metcalfe & Thorpe, 1992). Thus, we predicted that fish from small eggs or fish that emerge earlier will have similar morphology to wild fall migrants, which is correlated with the surface‐oriented phenotype, as shown by Unrein et al. (2017). We also conducted behavioral trials using fish from known egg size and emergence timing to evaluate their preference for location within the tank as well as overall activity. Based on the correlation between migration timing and vertical orientation in Unrein et al. (2017), our prediction was that fish from smaller eggs and those emerging earlier would show a significant preference for being near the surface of the tank.

## 2 MATERIALS AND METHODS

### 2.1 Fish source

On 23 September 2014, we collected eggs and milt from five hatchery broodstock spring Chinook Salmon females and males of the North Santiam River of the Willamette River basin, Oregon (Oregon Department of Fish and Wildlife). The hatchery broodstock was typical returning adults for production hatchery purposes and was approximately equivalent in size within each sex. We transported gametes to the Fish Performance and Genetics Laboratory in Corvallis, Oregon, where we artificially spawned fish in a single‐pair mating design. We chose a single‐pair mating design to evaluate maternal investment in egg size and general family‐level differences on future phenotypes without specifically evaluating if supposed differences are of maternal or paternal origin. The embryos were placed in a vertical incubation system, where multiple drawers (Heath trays) are used to incubate embryos. In our study, embryos from each family were incubated in separate Heath trays provided with constant 12°C pathogen‐free well water (7 L/min).

### 2.2 Establishing egg size and emergence timing groups

On 19 October 2014, we weighed a subset of 200 embryos (“eyed eggs”) from each female to determine overall size distribution and to establish upper and lower 20% boundaries for small and large egg groups. We weighed eggs individually using a microbalance (±0.0001 g) after removing excess water by dabbing eggs on clean paper towels. From 20 to 23 October 2014, we sorted out 400 small and 400 large embryos for each female based first on visual inspection and second with wet mass confirmation using the microbalance. We placed a subset of embryos in a shallow container with aerated water to conduct visual inspection of egg size and exchanged water frequently to maintain temperature. Once an embryo was selected as either small or large, the observer would remove it using egg tweezers, dab it on a clean paper towel to remove excess water, and then place it on a small tray on the microbalance to confirm size assignment. We then transferred each of the 400 small and 400 large embryos into separate containers and recorded the mass for each embryo. Throughout sorting, we periodically returned the sorted embryos to Heath trays marked by family and egg size category. Handling of salmonid embryos at this point of development is routine and has no ill effects on survival (Leitritz & Lewis, 1980).

On 23 October 2014, we transported the embryos for all 10 groups (small and large eggs for each family) in isolates to the Oregon Hatchery Research Center (OHRC). Here, we placed all 400 embryos from each group into one plastic hatching jar (H: 45.7 cm; D: 15.9 cm; V: 6L; Pentairaes.com, part #J30), for a total of 10 jars. Water enters the hatching jars at the bottom and exits over a spout at the top. This water pattern allows for egg rotation, and for hatched fish to swim up to the top and exit the jar volitionally. The hatching jars were supplied with flow‐through water (ambient Fall Creek; 1 L/min) and blocked from light with opaque coverings. To prevent emerging fish from being accidentally pushed out of the hatching jar with the flow of water, we added plastic filter media to each hatching jar after the embryos hatched.

Beginning in early December 2014, from the start of emergence, we monitored emergence daily and allowed the fish to emerge volitionally from the hatching jars. As the fish emerged, they fell into separated mesh collecting baskets (L × W × H: 34 × 23 × 18.5 cm), held adjacent in a flow‐through trough (L × W × H: 544 × 35.5 × 21.5 cm; flow: 18.9 L/min; mean temperature during emergence: 9.3°C). We collected the first 100 fish to emerge from each group after they exited the jar into each basket and placed them in marked baskets in another identical flow‐through trough. We then collected and discarded the next 150 emerging fish (number 101–250). We kept fish 251–300 in a separate basket to use to supplement the late‐emerging group as a result of possible mortality. Finally, we collected late‐emerging fish (number 301–400) and, after all fish emerged, supplemented these groups as needed using fish from the previous collection group (fish number 251–300). In the end, we had a total of four groups (100 fish each) for each of the five families: small egg early, large egg early, small egg late, and large egg late. We kept the groups separated in baskets in the flow‐through indoor troughs until we could mark the fish individually and combine across groups for each family.

On 19 February 2015, we used visible implant elastomer (Northwest Marine Technology, Inc.) to mark all fish using unique codes for each group (Leblanc & Noakes, 2012). After ensuring mark retention and accounting for any postmarking mortality, we combined the four groups from each family by equally dividing each group into two 0.9‐m‐diameter indoor tanks (volume: 356.9 L; Fall Creek water flow: 37.9 L/min), for a total of 200 fish in each tank, thereby creating a replicated common‐garden design for each family. We continued rearing fish until assessments were complete, which included taking digital photographs for morphometric analyses in March 2015 and conducting behavioral tests to evaluate preference for location in a tank in May 2015. Throughout rearing, we regularly monitored fish by taking monthly samples on fish sizes to determine overall growth and to adjust feed amounts. We fed fish commercially available diet (BioVita^®^; Bio‐Oregon) for the duration of rearing (initially ad libitum then at 1.5% body weight/day). Animal rearing, behavior experiments, and morphometric procedures were approved by the Institutional Animal Care and Use Committee at Oregon State University (ACUP #4289 and #4688).

### 2.3 Assessing morphology

When fish reached a mean fork length of 60 mm, approximately 2–3 months postemergence, we sampled fish to obtain images for morphometric comparisons. On 16–18 March 2015, we sampled 20 fish per treatment group from each replicate tank (80 fish per tank), for a total of 40 fish sampled per treatment group for each of the five families (800 photographs taken in total). To sample these fish and take the photographs, we first anesthetized fish to Stage III anesthesia (Tricaine Methanesulfonate, Western Chemical Inc., Ferndale WA, USA; hereafter, MS‐222, 50 mg/L buffered to pH 7.0 with sodium bicarbonate). Once anesthetized, we measured fork length (±1 mm) and took digital image of the left side of each fish (Nikon D7100 camera). One researcher was responsible for positioning the fish appropriately for morphometric photographs, while another researcher was responsible for taking each photograph. The camera was mounted to a fixed distance of approximately 30.5 cm above a clear Plexiglas tray containing the fish, water to maintain the fish's lateral position, and a ruler for a size standard. Additionally, the entire tray and camera were placed in a light box to produce uniform illumination without shadows and to reduce glare.

To compare body morphology, we used landmark‐based geometric morphometric analysis. We filtered out images of bent or tilted fish prior to conducting morphological comparisons to ensure differences in body shape were not due to artifacts of the imaging process. We used 15 landmarks frequently used for morphological comparisons in salmonids (Beeman, Rondorf, & Tilson, 1994; Billman et al., 2014; Tiffan & Connor, 2011). We digitized these landmarks using tpsDig2 (Rohlf, 2010) and ran a Generalized Procrustes Analysis (Gower, 1975; Rohlf & Slice, 1990) using R package *geomorph* (v. 3.0.3; Adams & Otárola‐Castillo, 2013). This analysis produces aligned Procrustes coordinates and a centroid size for each specimen that can be used in subsequent shape analyses.

### 2.4 Behavioral trials

From 12 May 2015 to 21 May 2015, we conducted behavioral trials to evaluate whether fish of known egg size and emergence timing showed behavioral differences in relation to preference to be near the surface and overall activity. We used four rectangular tanks (L × W × H: 58 × 30 × 40 cm) each set up with natural gravel substrate, an opaque back and side covering (yellow, similar to holding tank color), a mesh cover, and an overhead fluorescent light (13W). On the back covering, we marked the locations that divided the tanks horizontally into three equal compartments. We used static water, with water temperatures ranging from 10°C to 11.9°C during testing (consistent with rearing temperatures at time of testing). We recorded all trials (Hero and Hero2 GoPro camera) mounted ~100 cm in front of two tanks and recorded both tanks in one video. Because we ran two tanks in one video, we had two researchers present in all instances to prevent any time lag between tanks. Each of the dual tank setups was in a testing area behind black curtains to minimize disturbances during the testing period.

At the beginning of a trial, we removed two fish from one of the 10 rearing tanks (one fish for each test tank). We identified the tank (corresponding to family) and the location of the VIE mark and assigned the fish a number and testing tank. We wrote the date of the trial and fish numbers on a whiteboard and presented it at the beginning of each recording. In total, we recorded each trial for 35 min after we exited the testing area. We did not feed fish during the trial. After the trial time elapsed, we removed each fish from their testing tank, euthanized them with an overdose of buffered MS‐222 (250 mg/L), then measured their fork lengths and confirmed VIE marks. We performed three‐fourth water changes between each trial to maintain water quality. Altogether, we tested 10 fish per group for each female, for a total of 200 fish tested.

For each recording, we began a 15‐min acclimation period once the researchers were out of the testing area, followed by a 20‐min observation period. For the subsequent video analyses during the 20‐min observation period, we recorded the amount of time each fish spent in the top third portion of their tank, the number of line crosses into the top portion (measure of general activity level), and the amount of time spent inactive. We counted the total time a fish was inactive when it remained motionless while resting on the gravel substrate. Importantly, an inactive (motionless) fish is always associated with the bottom compartment and does not exhibit any body or fin movements. To be considered inactive, the fish needed to spend more than 1 min continuously inactive, and we included the initial minute toward the total inactive time. If the fish is associated with the gravel but continues to move their fins or undergo small movements without active swimming, then this fish is not considered to be inactive. The observer (JRU) was blind to the group identification of each fish.

### 2.5 Data analyses

To evaluate body shape differences due to egg size and emergence timing, we constructed a linear model using the *procD.lm* function in *geomorph*. In this model, we used the two‐dimensional set of Procrustes landmarks as the response variable and included egg size (a continuous variable, the mean egg mass for each female's small and large eggs), emergence timing (a categorical variable representing early and late designations for each female), and their interaction as the main effects. We included centroid size as a covariate in the model, to control for any size‐related effects on body shape. Finally, we also included female ID in the model, to control for possible shape variation as a result of family differences. This model function performs a MANCOVA with permutations (set to 1,000 permutations) to assess the relative amount of shape variation assigned to each factor (Adams & Otárola‐Castillo, 2013).

Because the small and large egg sizes for each female varied across females, we evaluated the effects of egg size and emergence timing within each female. Here, we ran five separate linear models again using *procD.lm* function in *geomorph*. We subset the data and used the two‐dimensional set of Procrustes landmarks for each of the five females as the response variables and included egg size (now a categorical variable representing the small and large egg groups within each female), emergence timing (a categorical variable representing early and late designations for each female), and their interaction as the main effects. We again included centroid size as a covariate in the model, to control for any size‐related effects on body shape. For all other analyses, we used R v. 3.2.3 (R Core Team, 2015). Specifically, we used linear mixed models (R package lme4 v. 1.1‐12; Bates, Maechler, Bolker, & Walker, 2015) to estimate the effect of mean egg size, emergence timing, and their interaction on the time spent in the top portion of the tank and the time spent inactive, dropping the interaction whenever not significant. For these two models, we log transformed the time spent at the top (+ 1s for each value) and square root transformed the time spent inactive to improve normality of the model residuals based on a Box‐Cox analysis. We used quantile–quantile and residuals‐versus‐fitted diagnostic plots to visually inspect for normality and homogeneity of variance. Finally, we included body length as a covariate with emergence timing and included family and testing day as fixed effects in these two models. For number of line crossings into the top portion of the tank, we used a negative binomial generalized linear model (glm.nb; R package *MASS* v. 7.3‐45) and again included mean egg size, emergence timing, and their interaction, as well as a length covariate and family and testing day as fixed effects in the model.

To evaluate size and growth differences across groups, we ran multiple linear mixed models using the log of the fork length as the response variable and the random effect of family origin in each model. First, we compared body size at emergence, at the time we took morphometric photographs, and when we ran behavioral trials. At emergence, we ran two models to look at differences in fish size within both the early and late emergence groups, including only mean egg size as the fixed effect for each model. To compare fish length at the later sampling event, we ran a linear mixed model with mean egg size and emergence time category as the fixed effects. Finally, to evaluate growth differences from emergence time through morphometric sampling, we included sampling date (four sample events), mean egg size, and emergence timing, along with the interactions between sampling date and mean egg size as well as sampling date and emergence timing in the model.

## 3 RESULTS

### 3.1 Egg size

**Figure 1:**
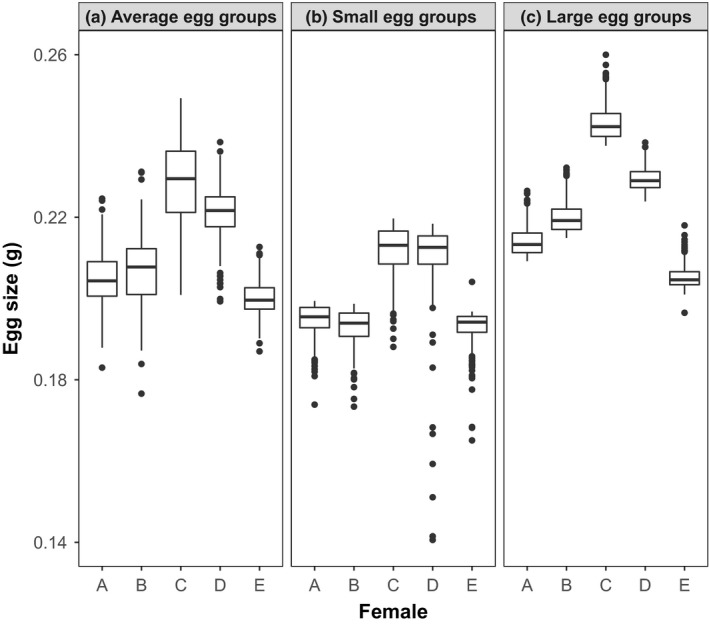
Variation in egg size (in g) across five female Chinook Salmon (*Oncorhynchus tshawytscha*). (a) Average variation in egg size based on a random sample of 200 eggs from each female. After establishing egg size distribution within females, eggs were sorted into small and large egg groups based on mass. Variation in egg size shown within (b) small and (c) large eggs groups for each female (*N* = 400 eggs per small and large egg group per female). All eggs were weighed individually, after removing excess water, using a microbalance with 0.0001 g accuracy. Box plots show median, first and third quartiles, 95% confidence intervals, and outlier data points

Across the five females used in our study, we found significant differences in egg size (*F*_4, 994_ = 500.77, *p* < .0001; Figure 1a, Table 1). All pairwise comparisons were significantly different (Tukey HSD: *p* < .0001) except for a marginal difference between female A and B (*p* = .08). After size sorting for small and large eggs for each female, we observed overlap in size categories (Figure 1b,c, Table 1). As such, we decided to use egg size as a continuous variable in subsequent analyses. Further, because the difference between groups of eggs was not consistent, we used mean egg size for each group as opposed to a rank.

**Table 1:** Mean egg size by female for overall average egg size measured (*N* = 200 each) and after separating eggs into small (*N* = 400 each) and large (*N* = 400 each) categories in Chinook Salmon (*Oncorhynchus tshawytscha*)

Female	Mean egg size (g)^a^ ± *SD*		
	Average	Small	Large
A	0.2051 ± 0.007	0.1948 ± 0.004	0.2142 ± 0.004
B	0.2071 ± 0.009	0.1931 ± 0.004	0.2199 ± 0.004
C	0.2288 ± 0.010	0.2119 ± 0.006	0.2431 ± 0.004
D	0.2214 ± 0.007	0.2119 ± 0.009	0.2295 ± 0.003
E	0.2000 ± 0.004	0.1931 ± 0.004	0.2051 ± 0.003

a Measurements taken using a microbalance with 0.0001 g accuracy.

### 3.2 Morphometrics

**Figure 2:**
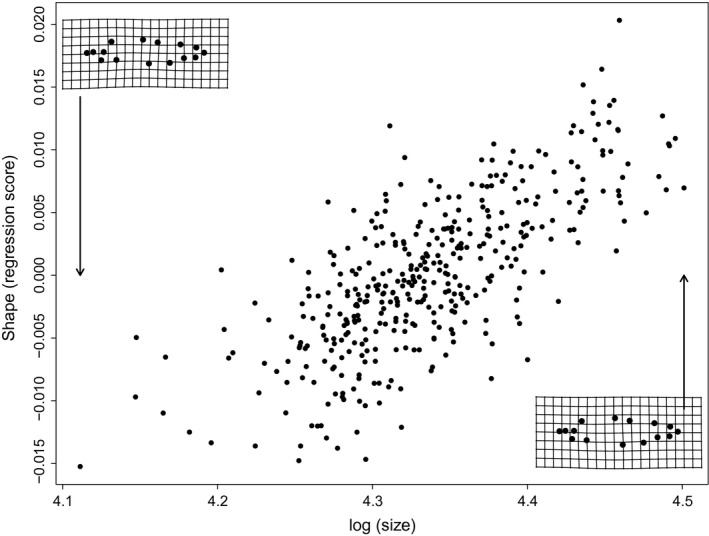
The relationship between body shape and body size in juvenile Chinook Salmon (*Oncorhynchus tshawytscha*). Analysis is a Procrustes regression to evaluate size covariation with body shape, using *ProcD.allometry* function in R package *geomorph*. Thin plate splines deformation grids show the deviation from the overall consensus shape, at the extremes

**Figure 3:**
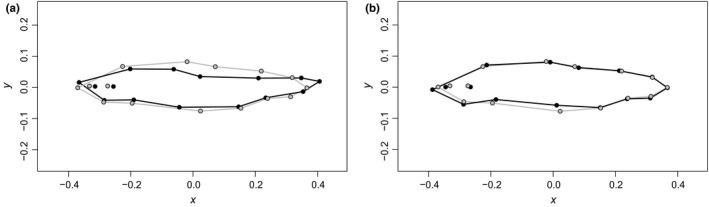
Morphological depictions of juvenile Chinook Salmon (*Oncorhynchus tshawytscha*), magnified by 10× to aid visualization. (a) Mean shape differences between the smallest (gray) and largest (black) egg size groups. Because egg size was used as a continuous variable, the mean shapes presented here reflect only the fish from smallest egg group (Female B; mean egg size: 0.19309 g) and the largest egg group (Female C; mean egg size: 0.24315 g). (b) Mean shape differences between early (gray) and late (black) emergence groups, across all females. Data are standardized shape coordinates based on landmark data in two‐dimensional space from Generalized Procrustes Analysis

There was a significant effect of body size on body shape (Procrustes multivariate analysis of covariance (MANCOVA): *F*_1, 381_ = 33.545, *p* = .001; Table 2; Figure 2), but this relationship did not vary with egg size, emergence timing, or their interaction (Table 2). Controlling for body size and family origin, body shape was significantly correlated with both egg size (*F*_1 ,381_ = 14.338, *p* = .001; Table 2) and emergence timing (*F*_1, 381_ = 10.656, *p* = .001; Table 2). Fish from small eggs had deeper heads, deeper and shorter caudal peduncles, and deeper bodies compared to fish from large eggs (Figure 3a). Additionally, fish that emerged earlier had shorter heads and deeper bodies compared to fish that emerged later (Figure 3b).

**Table 2:** MANCOVA using Procrustes landmarks to evaluate body shape variation in juvenile Chinook Salmon (*Oncorhynchus tshawytscha*)

	*df*	SS	MS	*F*	*p* a
**Egg size**	**1**	**0.0028**	**0.0028**	**14.338**	**.001**
**Emergence time**	**1**	**0.0021**	**0.0021**	**10.656**	**.001**
**Log centroid size**	**1**	**0.0066**	**0.0066**	**33.545**	**.001**
**Female**	**4**	**0.0196**	**0.0049**	**24.854**	**.001**
Egg size × Emergence time	1	0.0002	0.0002	0.759	.635
Egg size × Log centroid size	1	0.0003	0.0003	1.456	.138
Emergence time × Log centroid size	1	0.0003	0.0003	1.442	.146
Egg size × Emergence time × Log centroid size	1	0.0001	0.0001	0.689	.704
Residuals	381	0.0753	0.0002		
Total	392	0.1074			

Significant effects are shown in bold.

a *p* values based on 1,000 permutations using the *ProcD.lm* function in R package *geomorph*.

**Figure 4:**
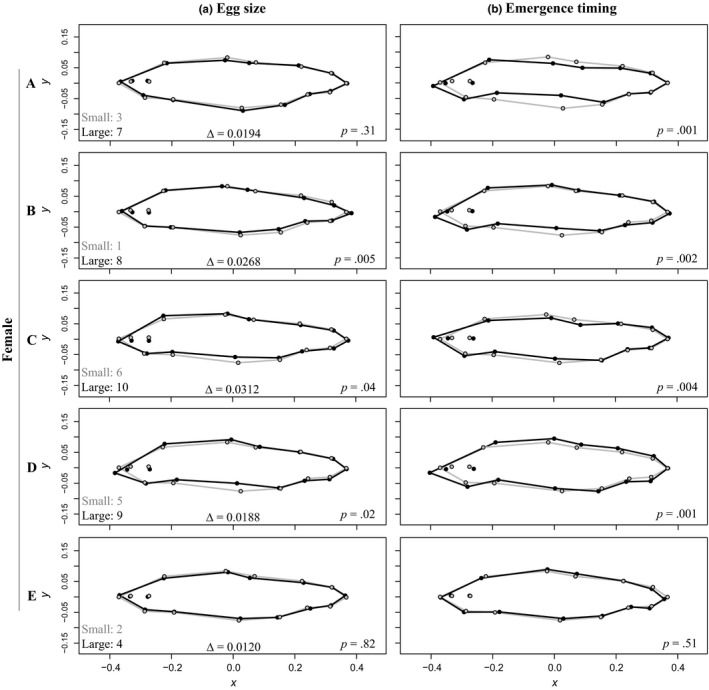
Morphological depictions of juvenile Chinook Salmon (*Oncorhynchus tshawytscha*), separated by female (A–E). Shape differences are magnified by 10× to aid visualization. (a) Mean body shape variation based on egg size. Within female, egg size was used as a categorical variable: small (gray) and large (black) eggs. The overall rank (out of 10 groups across females) is given for each egg group, along with the mean difference in egg size (∆) between individual females small and large eggs at the time of sorting. (b) Mean body shape differences between early (gray) and late (black) emergence groups. Data are standardized shape coordinates based on landmark data in two‐dimensional space from Generalized Procrustes Analysis. *p* values provided with each morphological depiction are based MANCOVAs run for each female with 1,000 permutations using the *ProcD*.lm function in R package *geomorph*

When we looked at the effects of egg size and emergence timing within each female separately, we found that body shape was correlated with egg size for three of the females, and with emergence timing for four of the females (Figure 4). Because mean egg size for the small and large egg groups varied across females, we were most interested in the within‐female effect of egg size on body shape. For egg size, offspring of females B, C, and D had significantly different body morphologies. Two of these females (B and C) had that greatest size difference between the small and large eggs (difference in mean egg size between groups: Female B = 0.0268 g, Female C = 0.0312 g). Conversely, females A and E did not have significant differences in body shape between egg groups and had the smallest difference in mean egg size between their small and large groups (Female A = 0.0194 g, Female E = 0.0120 g). This suggests that the effect of egg size on body shape is an absolute effect, as opposed to relative.

### 3.3 Behavioral trials

**Figure 5:**
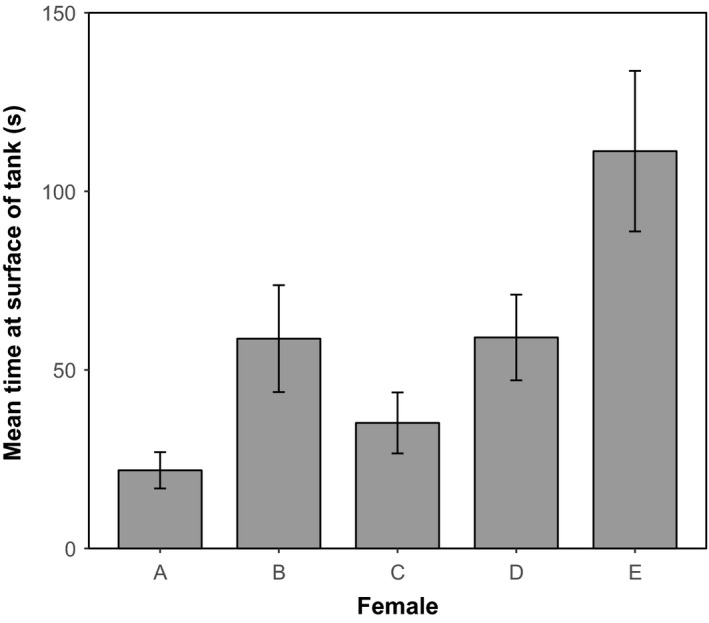
Meantime (s) ± *SE* that juvenile Chinook Salmon (*Oncorhynchus tshawytscha*) spent in the top third portion of the tank separated by family origin (Female). The total duration of the behavioral trial was 1,200 s. Raw data shown; model included variance attributed to egg size, emergence timing, and testing day

For the time spent in the top third portion of the tank, the interaction between egg size and emergence timing was not significant (*F*_1, 185_ = 0.51, *p* = .48) and therefore removed to evaluate the main effects of egg size and emergence time. There was a significant effect of the length covariate with emergence timing (*F*_1, 186_ = 4.23, *p* = .04), such that small late‐emerging fish in particular, and late‐emerging fish in general (*F*_1, 186_ = 4.53, *p* = .03), spent more time in the top portion of the tank. The time spent in the top portion of the tank was not significantly related to egg size (*F*_1, 186_ = 0.67, *p* = .41). However, there was a significant effect of family in the amount of time spent near the top of the tank (*F*_4, 186_ = 5.22, *p* = .0005; Figure 5). Specifically, the offspring from female E spent significantly longer in the top third portion of the tank compared to offspring from females A, B, and C (all *p* < .02).

For number of line crosses into the top portion of the tank, we again showed no significant interaction between egg size and emergence timing (glm.nb: χ^2^ = 0.03, *p* = .96), and a significant effect of the length covariate with emergence timing (χ^2^ = 2.26, *p* = .02). Small late‐emerging fish in particular, and late‐emerging fish in general (χ^2^ = 2.38 *p* = .02), crossed into the top portion of the tank more frequently. There was no significant effect of egg size (χ^2^ = 0.40, *p* = .69) on the number of line crosses. In relation to the time spent near the surface of the tank, offspring from female E crossed significantly more often into the top portion of the tank compared to offspring from all other females (all *p* < .05 ).

Similarly, the interaction between egg size and emergence timing on the time spent inactive in the tank was not significant (*F*_1, 185_ = 0.22, *p* = .64). There was a significant effect of the length covariate with emergence timing (*F*_1, 186_ = 4.17, *p* = .04), such that small early emerging fish in particular, and early emerging fish in general (*F*_1, 186_ = 4.81, *p* = .03), spent more time inactive. There was also no significant effect of egg size (*F*_1, 186_ = 1.00, *p* = .32) on the amount of time spent inactive. However, the offspring from female D were significantly more active than the offspring of female A (*p* = .04) and B (*p* = .01).

### 3.4 Size and growth

Fish size was positively influenced by mean egg size within both early (*F*_1, 297.8_ = 29.7, *p* < .0001) and late (*F*_1, 297.65_ = 7.78, *p* = .006) emergence groups, such that larger eggs produced larger fish at emergence.

**Figure 6:**
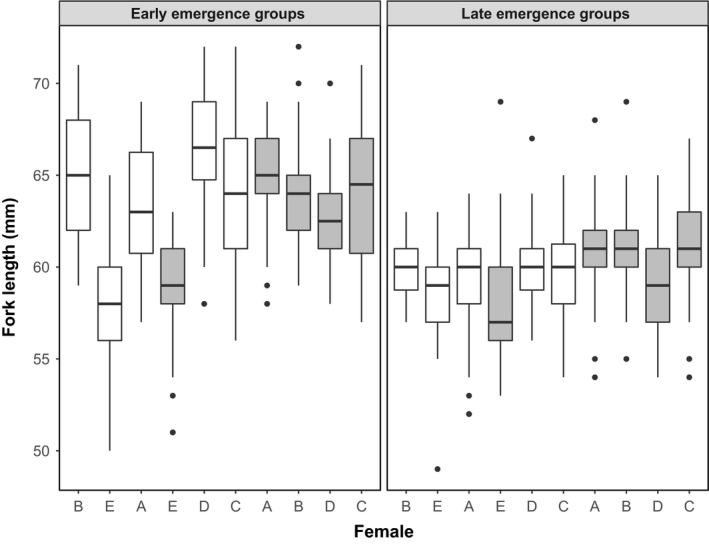
Fork length (in mm) of juvenile Chinook Salmon (*Oncorhynchus tshawytscha*) at the time of morphometric analyses. On *x*‐axis, box plots are plotted in order of smallest to largest mean egg size for both the early and late emergence groups. Axis shows which female (A–E) and relative egg size within each female (small egg = white, large egg = gray). Box plots show median, first and third quartiles, 95% confidence intervals, and outlier data points (*N* = 30 fish for each group represented)

At the time we took the photographs for morphometric analyses, fish that emerged early were significantly larger than fish that emerged late (*F*_1, 790.01_ = 228.3, *p* < .0001; mean ± *SE* for Early: 63.14 ± 0.20 mm; Late: 59.75 ± 0.14 mm; Figure 6). The initial mean egg size for each group did not significantly influence fish size at the time of measurement (*F*_1, 767.32_ = 1.19, *p* = .28; Figure 6). We found similar differences in fish size observed at the end of the behavioral trials as we did for morphometric analyses. Accounting for the random effect of family, fish that emerged early were significantly larger than fish that emerged late (*F*_1, 188.83_ = 55.94, *p* < .0001; mean ± *SE* for Early: 79.93 ± 0.67 mm; Late: 73.60 ± 0.52 mm). Fish sizes were not significantly different across mean egg size (*F*_1, 74.33_ = 0.09, *p* = .77).

For our growth model, there was a significant interaction effect between sampling date and mean egg size (*F*_1, 2287_ = 38, *p* < .0001). Fish that originated from smaller eggs had a significantly greater increase in fish size over time. There was no significant difference in growth rate between early and late‐emerging fish (*F*_1, 2287_ = 0.5, *p* = .48).

## 4 DISCUSSION

In the Willamette River basin, USA, fall and spring wild juvenile migrants differ in body morphology (Billman et al., 2014), and we found that variation in egg size appears to be correlated with similar morphological differences. As we predicted, body morphology of fish from small eggs was more similar to the deep‐bodied fall migrant, while fish from large eggs had a body morphology consistent with that of fusiform spring yearling migrants. Our results also showed that this pattern may be based on absolute egg size, as opposed to relative egg size within each family. Additionally, fish that emerged earlier also had deeper bodies compared to late‐emerging fish. Contrary to our prediction, fish that emerged later showed a greater preference to be near the surface of the tank, and we found no association with egg size. We evaluated movement and orientation in a tank as a possible link to the behavioral surface‐ and bottom‐orientation phenotypes that may predict fall migration in juvenile spring Chinook Salmon of the same river (Unrein et al., 2017). Throughout our study, it was evident that family origin accounted for significant differences in egg size, body morphology, and behavior.

Egg size is largely a maternal effect that can have important consequences on the subsequent expression of life history traits. Juvenile salmonids that hatch from smaller eggs often have a faster subsequent growth rate in comparison with their counterparts that hatched from larger eggs (Eldridge et al., 1982; Heath et al., 1999; Leblanc, 2011; Segers et al., 2012; Valdimarsson et al., 2002). We show the same pattern in our study, where fish that hatched from smaller eggs grew at a faster rate than fish that hatched from larger eggs. Early growth rate influences the timing of juvenile downstream migration. Fish with faster growth rates are more likely to migrate as subyearlings in both Chinook Salmon (Perkins & Jager, 2011) and Atlantic Salmon (Metcalfe & Thorpe, 1992). In both studies, this commitment to a migratory life history was suggested to occur soon after emergence. Based on the differences in growth rate between smaller and larger egg groups, and the similar body morphologies to fall and spring migrants, respectively (Billman et al., 2014), our study provides further support that early growth rate may be contributing to subsequent migratory timing in juvenile Chinook Salmon.

Compared to the study by Perkins and Jager (2011) and Metcalfe and Thorpe (1992), we suggest that the decision on when to migrate may be occurring even prior to emergence, based on egg size, and may therefore have stronger genetic, epigenetic, and maternal condition influences than previously thought. The decision “thresholds” or “switchpoints” for juveniles migrating downstream can also show additive genetic variance and genetic variation in life history reaction norms (Hutchings et al., 2007; Piché, Hutchings, & Blanchard, 2008; Roff, 2011; Tomkins & Moczek, 2009). Our study suggests variation in egg size may be an important determinant of future phenotypic expression, perhaps correlated with migratory timing in juvenile salmon. We suggest that the decision thresholds may be affected by differences in early growth rates (Beckman et al., 1998; Gross, 1987).

We had predicted that fish that emerged earlier would have similar morphology to the fall migratory phenotype (Billman et al., 2014; Metcalfe & Thorpe, 1992). That is, early emerging fish are generally larger and may therefore surpass a size/growth threshold at a critical point in development that may lead to earlier migration. Fish that emerged earlier were larger both at emergence and later in development, but this did not appear to have a strong influence on body morphology associated with downstream migration timing. The only similarity in body morphologies between early emerging and early migrating fish was a slightly deeper body shape. Instead, the effect of emergence timing may be influenced more by environmental differences at emergence. Importantly, early and late‐emerging fish may still have had similar growth rates as a result of their rearing environment. If natural differences in early growth rate are strong predictors of migration timing, this may explain why mean egg size rather than emergence timing appeared to be driving the pattern in body morphology associated with migration timing.

In our behavioral assessment, we did not find a significant effect of egg size on the time spent near the surface of the tank, the number of line crosses into the surface of the tank, and general inactivity. However, we did find that late‐emerging fish, especially those of smaller length, spent more time in the top portion of the tank, crossed into the top portion more frequently, and were generally more active. It is important to state here that our behavioral trial was designed to evaluate general behavioral patterns and tank orientation. This was not designed in the same fashion that Unrein et al. (2017) observed distinct surface‐ and bottom‐oriented phenotypes. Additionally, the acute stressor applied by individually testing fish in a novel tank environment may have impacted general behavioral patterns (e.g., Noakes & Jones, 2016; Schreck & Tort, 2016). Importantly, we did find significant family differences in all behaviors assessed. These family differences in behavior may be overriding any general patterns based on egg size in this assessment. The offspring of one female in particular (Female E) spent significantly more time than the other families near the surface of the tank. Interestingly, both the small and large egg categories from this female were some of the smallest egg sizes seen among the five females used in this study (ranked 2nd and 4th smallest, respectively, of 10 possible ranks). Just as fish from small eggs often have increased early growth rate, Sundström, Devlin, Johnsson, and Biagi (2003) have shown that increased growth rate may lead to an increase in a preference to be near the surface in transgenic Coho Salmon.

The results of our study show that subtle differences in early life history characteristics can contribute to future variation in morphological and behavioral phenotypes. Our results also suggest that these early life history differences may be correlated with different migration timing in a population of spring Chinook Salmon. If morphology is a predictor of migratory behavior, then our study provides support for using early life history characteristics as predictors of migration.

## AUTHOR CONTRIBUTIONS

All authors contributed to conception and design of the work. KMC led data collection, analyzed the behavioral and morphological data, interpreted the data, and drafted and revised the manuscript. JRU contributed to data collection and behavioral observations, analyzed the morphometric data, interpreted the data, and provided revisions on the manuscript. HAS contributed to data collection for morphometric analysis, interpreted the data, and provided revisions on the manuscript. CBS and DLGN both contributed to data interpretation and provided critical revisions on the manuscript. All authors approved the final version of this submitted manuscript.

## CONFLICT OF INTEREST

None declared.
